# 18.4 CHARACTERIZING LARGE-SCALE NETWORK DYSCONNECTIVITY IN SCHIZOPHRENIA: MODELING ALTERED E/I BALANCE

**DOI:** 10.1093/schbul/sby014.073

**Published:** 2018-04-01

**Authors:** Alan Anticevic

**Affiliations:** Yale University

## Abstract

**Background:**

Neuropsychiatric disorders alter the structure and function of neural circuits and distributed neural networks, which leads to profound behavioral disability such as working memory impairment. Non-invasive neuroimaging tools have matured to reliably detect neural systems-level disturbances in neuropsychiatric disorders. However, mechanistic mapping from neural circuit pathology to abnormal behavior remains out of reach for most psychiatric conditions. We focus on the emerging field of ‘computational psychiatry’ to close these gaps from the perspective of E/I imbalance in schizophrenia with an emphasis on understanding large-Scale network dysconnectivity in schizophrenia.

**Methods:**

First, we apply computational microcircuit models to understand cognitive and neural system-level disturbances in schizophrenia as a function of altered E/I balance. In turn, the focus is placed on pharmacological neuroimaging as a powerful causal tool to probe neural circuit perturbations. Specific recent neuroimaging studies are discussed that use the NMDA receptor antagonist, ketamine, to probe glutamate synaptic dysfunction associated with schizophrenia. We leverage resting-state neuroimaging advances to inform biomarker development for network alterations in schizophrenia given its ease of data collection and lack of task-based confounds.

**Results:**

Pharmacological and clinical results implicate alterations in cortico-striato-thalamic circuits, which might constitute a final common pathway of neural system disturbances in schizophrenia. Results indicate that this functional marker appears in at-risk states, chronic patients, and following pharmacological manipulations following ketamine. Computational simulations implicate altered E/I balance in cortical microcircuits as a parsimonious upstream mechanism.

**Discussion:**

The combined use of biophysical computational modeling, extended to large-scale neural system simulations, has proven particularly powerful to draw inferences about neural circuit alterations that may be ‘driving’ the resting-state dysconnectivity and cognitive deficits in schizophrenia. In summary, present data illustrates a framework for linking experimental studies in humans with computational models and pharmacological probes will advance to effort to bridge cellular, systems, and clinical neuroscience approaches to psychiatric disorders.

